# EGFR-Targeting as a Biological Therapy: Understanding Nimotuzumab's Clinical Effects

**DOI:** 10.3390/cancers3022014

**Published:** 2011-04-18

**Authors:** Rolando Perez, Ernesto Moreno, Greta Garrido, Tania Crombet

**Affiliations:** Center of Molecular Immunology, P.O. Box 16040, Havana 11600, Cuba; E-Mails: emoreno@cim.sld.cu (E.M.); greta@cim.sld.cu (G.G.); taniac@cim.sld.cu (T.C.)

**Keywords:** EGFR, Nimotuzumab, cancer treatment, targeted therapy, biological therapy

## Abstract

Current clinical trials of epidermal growth factor receptor (EGFR)-targeted therapies are mostly guided by a classical approach coming from the cytotoxic paradigm. The predominant view is that the efficacy of EGFR antagonists correlates with skin rash toxicity and induction of objective clinical response. Clinical benefit from EGFR-targeted therapies is well documented; however, chronic use in advanced cancer patients has been limited due to cumulative and chemotherapy-enhanced toxicity. Here we analyze different pieces of data from mechanistic and clinical studies with the anti-EGFR monoclonal antibody Nimotuzumab, which provides several clues to understand how this antibody may induce a biological control of tumor growth while keeping a low toxicity profile. Based on these results and the current state of the art on EGFR-targeted therapies, we discuss the need to evaluate new therapeutic approaches using anti-EGFR agents, which would have the potential of transforming advanced cancer into a long-term controlled chronic disease.

## 1. Introduction

Epidermal growth factor receptor (EGFR)-targeted therapies have been extensively evaluated in the clinic [1,2], and several EGFR-targeting products have been registered worldwide for the treatment of different tumor localizations [2]. However, the clinical benefit of these products in advanced cancer patients, in terms of median overall survival, has been limited so far (Table 1). Different factors may contribute to restrict the effect of anti-EGFR drugs on survival, for example, lack or escape from EGFR-addiction in tumors through mutations in other genes (e.g., K-Ras mutations) and pathways [3]. Remarkably, no K-Ras mutations have been found in colorectal cancer patients responding to Cetuximab or Panitumumab treatment in different studies [4-8]. Furthermore, studies conducted in non-small-cell lung cancer patients indicate that K-Ras mutation may be a negative response predictor to erlotinib and gefitinib [9,10].

The limited effects on survival of EGFR antagonists might nonetheless be also a consequence, at least partially, of administering EGFR-targeting therapies in a non-optimal way. Induction and maintenance phases, treatment after early progression, combination therapies and response predictor biomarkers are important issues currently in debate to optimize the clinical benefit of these therapies. On the other hand, we are lacking in differentiation strategies for individual EGFR antagonists, which could eventually improve their clinical benefit in different patient niches. In this paper, taking the antibody Nimotuzumab as a case study, we would like to share our views regarding clinical implementations of EGFR-targeted therapies that aim to a long-term control of the advanced cancer disease.

## 2. The Current Paradigm: Clinical Efficacy Is Bound to Cytotoxicity

There is an increasing understanding within the clinical researchers' community that the therapeutic endpoints of molecularly targeted agents should be revised, since neither toxicity nor tumor shrinkage are necessarily adequate surrogates to evaluate their clinical efficacy [22,23]. Furthermore, in some tumors like high-grade glioma, the use of traditional, imaging-based endpoints such as overall radiographic response and progression-free survival has become problematic due to pseudoprogression, observed with different types of therapy, and pseudoresponse, reported for anti-angiogenic agents such as Bevacizumab [24].

Current clinical trials of EGFR-targeted therapies are, nevertheless, still guided by a classical approach coming from the cytotoxic paradigm. The predominant view is that the clinical efficacy of EGFR antagonists correlates with skin rash toxicity, as documented for most EGFR-targeted agents [2,25], and induction of objective clinical response [13]. This therapeutic approach, however, has shown limitations in the clinical practice, where cumulative and chemotherapy-enhanced toxicity has impaired the chronic use and combination therapies [26]. A relevant question here is whether toxicity is really welded to the clinical efficacy of EGFR-targeting drugs, assessed in terms of overall survival. If this is not the case, then EGFR-antagonists would need to be evaluated in the clinic using other criteria, different from those applied for cytotoxic drugs.

Novel criteria, designated as immune-related response criteria (irRC), have been recently proposed based on the experience with ipilimumab in patients with advanced melanoma [27]. They represent an extension of the response evaluation criteria in solid tumors (RECIST) or WHO criteria, which were developed to standardize the efficacy evaluation of cytotoxic drugs, to be applied to immunotherapeutic agents having predominantly a cytostatic effect [23]. An irRC major contribution is the definition of new clinical response patterns, involving “mixed responses”, pseudoprogression and early progression phenomena, which correlate with overall survival, thus providing a useful tool for a more accurate assessment of the efficacy of novel immunotherapeutic agents [27].

## 3. Diverging from the Cytotoxic Paradigm in Anti-EGFR Therapies

### 

#### Relevance of EGFR-signaling for tumor biology

Self-sufficiency in growth signals is one of the six hallmarks of cancer postulated by Hanahan and Weinberg [28]. The overexpression of EGFR in human breast cancer and its relation to bad prognosis prompted us, more than twenty years ago, to extend the concept of tumor hormone-dependence to growth factors such as EGF [29]. On the other hand, HER1 (EGFR) oncogene activation results in EGFR overexpression in epithelium-derived tumors, leading to a relaxation of the growth factor dependency. Our knowledge on the role of EGFR in the cell biology has been further expanded with the emergence, in recent years, of new experimental data demonstrating an interplay between oncogene signaling pathways, tumor metabolic re-programming and cancer-related inflammation [30,31]. In consequence, our understanding of EGFR-targeting therapies should also be expanded beyond the cytotoxic paradigm, in correspondence with the pleiotropic nature of the receptor signaling network. In the following sections we discuss recent findings from mechanistic and clinical studies of Nimotuzumab (also known as h-R3), a humanized anti-EGFR monoclonal antibody (mAb) [32], which provide a different perspective on EGFR-targeting therapies.

### 3.1. Clinical Experience with Nimotuzumab

#### A low toxicity profile

The therapeutic effects observed for Nimotuzumab in patients with head and neck squamous cell carcinoma [33-35], pancreatic cancer [36], non-small cell lung cancer [37] and glioma [38-40] have been characterized by the induction of a long-term stable disease with a very low toxicity profile, in contrast to other anti-EGFR agents [32,41]. Even though Nimotuzumab produces a downstream inhibition of the EGFR signaling pathway in normal skin cells, the characteristic lymphocytic infiltrates, folliculitis or perifolliculitis induced by other EGFR inhibitors have not been observed [32].

Table 2 summarizes the published clinical results with Nimotuzumab. Although conclusive assessment of clinical efficacy is pending on completion of the currently ongoing phase III clinical trials, multiple evidences of clinical benefit have been obtained so far. Paradoxically, the increase in overall patient survival produced by Nimotuzumab is not necessarily accompanied by objective clinical responses. In fact, in some patients tumors did not regress, but became “frozen” for months and even for years. The clinical data gathered in the most recent years from more than 9,000 patients, in open populations with different ethnic characteristics [42], reinforce the conclusions extracted from the clinical trials on Nimotuzumab's safety profile.

#### Long-term chronic treatment

**Figure 1.**
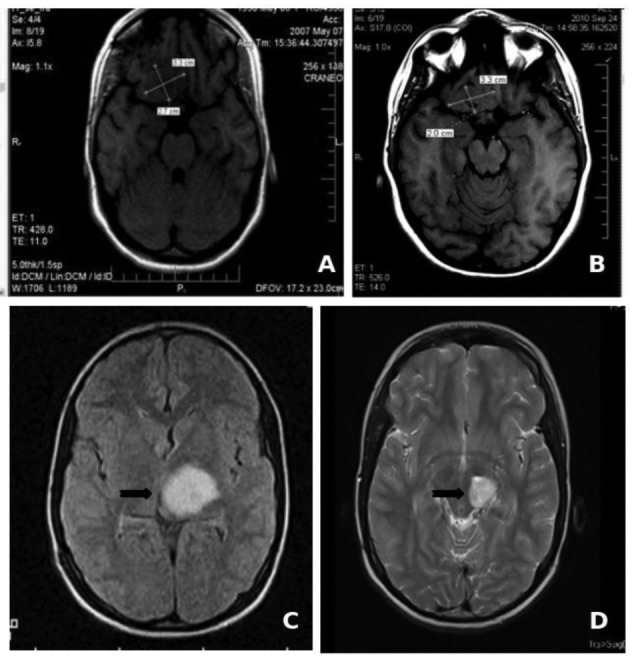
Conventional MRI imaging of Nimotuzumab long-term treated tumors for two pediatric pontine glioma patients, showing a prolongued stable disease (SD) after more than 100 doses of Nimotuzumab. Patient AFR: (A). May 2007, tumor diameters (TD): 3.3 cm × 2.2 cm; (B). September 2010, TD: 3.3 cm × 2.0 scm. Patient GRA: (C). December 2007; (D). April 2010, black arrows point to tumor images.

The low toxicity profile shown by Nimotuzumab has allowed its use in prolonged treatments, lasting several months, and even years in many cases. About two thirds of the 600 patients that have been treated in Cuba since 2002 have received more than six doses of the antibody (200 mg for adult, 150 mg for children), including about 50 patients that have been treated with more than 30, bi-weekly doses during more than one year [50]. In particular, two children with brain stem glioma tumors have received more than 100 doses of Nimotuzumab, continuously for more than three years, without showing adverse effects (Figure 1). It is worth noting that the frequency of adverse events (limited to grade 1 or 2) observed in these patients did not increase with drug exposure. From this clinical experience we have obtained important evidences on the impact of chronic treatment in disease stabilization and increase of overall survival in advanced cancer patients.

Perhaps such long-term treatments would be feasible also with other anti-EGFR antibodies, if the dosage and treatment schedules were adjusted to avoid a strong cumulative toxicity. As for Nimotuzumab, lowering the toxicity might result also in a reduction of the frequency of objective clinical responses. Currently, anti-EGFR antibodies are administered in large doses (hundreds of milligrams) following the paradigm developed for cytotoxic drugs, whereas biological therapies would require different evaluation criteria to define the optimal dosage and administration schedule. The relevance of chronic treatment with Nimotuzumab on disease control is currently being assessed in controlled clinical trials (e.g., NCT00561990 and NCT00753246, [51]) comparing a maintenance phase with the antibody *versus* the best supportive care.

### 3.2. Mechanisms of Action behind Nimotuzumab's Low Toxicity Profile

**Figure 2.**
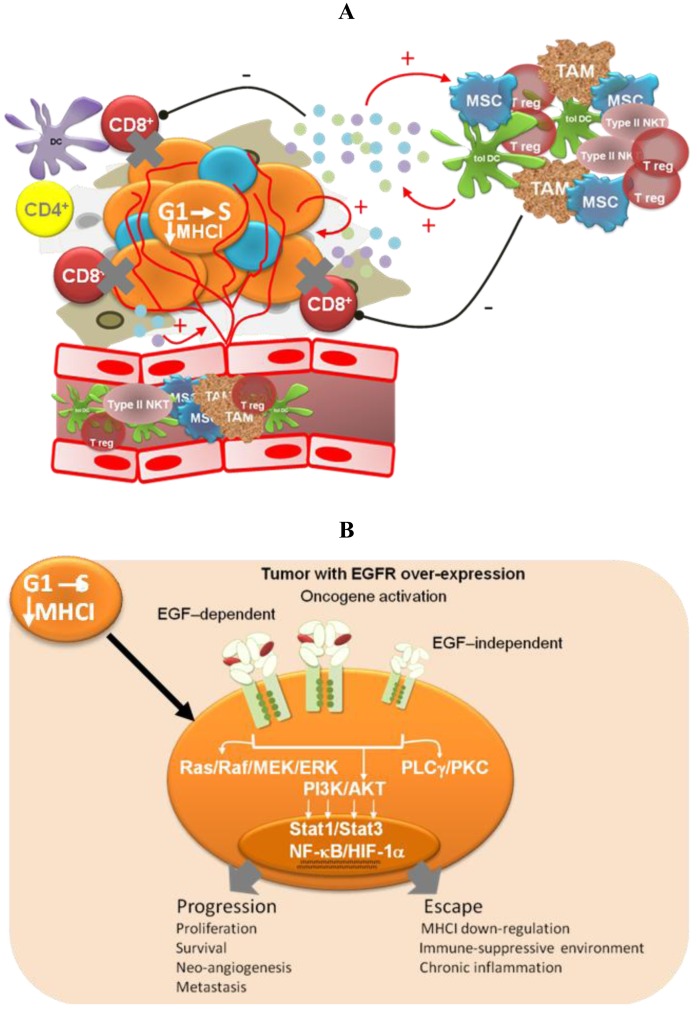

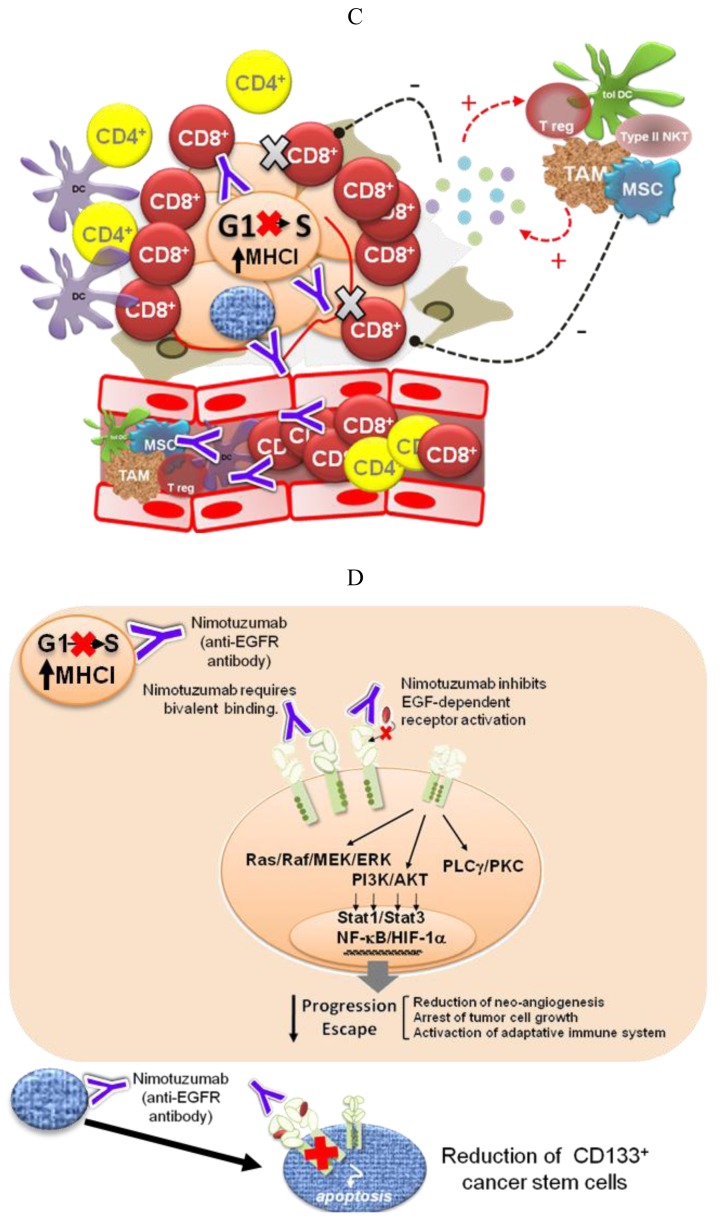
(**A**). Strategies exploited by tumors to progress and evade the immune response. The picture shows developing tumor cells (orange), cancer stem cells (blue) as well as underlying stroma and nontransformed cells (gray). The different lymphocyte populations are labeled, while the small blue, green and violet, circles represent immune-suppressive factors, chemokines and pro-inflammatory cytokines; (**B**). Effects of EGFR activation on tumor development; (**C**). Treatment with an anti-EGFR mAb would induce a biological control of tumor growth and downregulation of the immune-suppressive inflammatory environment. The drawing shows tumor cells arrested in the G0/G1 phase (light orange) and apoptotic cancer stem cells (rough blue), resulting from treatment with Nimotuzumab (violet). For the rest of the elements in the panel, the same labeling scheme and color code as in (a) was applied; (**D**). Nimotuzumab's mechanisms of action.

Different pieces of experimental and modeling data, gathered in recent years, support four complementary mechanisms to explain the low degree of adverse effects and the long-term disease stabilizations observed for Nimotuzumab in the clinic (Figure 2).

#### Intermediate affinity for EGFR and need of bivalent binding

The development of antitumor antibodies has been driven by the assumption that higher-affinity mAbs (having a dissociation constant (KD) in the nanomolar order or even lower) will have superior tumor targeting and efficacy properties. It has been shown, however, that antibodies with very high affinity have a lower penetration into solid tumors [52]. On the other hand, when the antigen targeted by the high-affinity mAb is not tumor-specific, large amounts of the antibody are retained in normal tissues. The two FDA-approved anti-EGFR antibodies, Cetuximab and Panitumumab, are high-affinity mAbs, with KD values for their monovalent Fab fragments of 2.3 × 10^−9^ M [53] and 5 × 10^−11^ M [54], respectively. Nimotuzumab, in contrast with these two antibodies, has a lower, “intermediate” affinity (KD = 2.1 × 10^−8^ M [55]).

Based on a mathematical model, a few years ago we put forward the hypothesis that antibodies with intermediate affinities, like Nimotuzumab, would have a higher ratio of accumulation in tumors (showing higher EGFR expression levels) with respect to normal tissues, as compared to high affinity antibodies [43]. Two recent reports [56,57] give support to this hypothesis. They show that binding of Nimotuzumab and subsequent inhibition of the EGFR phosphorylation are detected only for tumor cells lines with medium or high levels of EGFR expression (10^4^ receptors per cell or higher). Furthermore, binding of Nimotuzumab Fab fragments was detected only for A431 cells, having the highest EGFR expression level, whereas Cetuximab Fab fragments bound also to tumor cells with lower EGFR expression levels [57]. Thus, these results sustain also the idea that Nimotuzumab requires bivalent attachment for binding to tumor cells having a surface density of EGFR molecules above certain threshold. On the other hand, Akashi and coworkers reported that the *in vitro* and *in vivo* effect of Nimotuzumab combined with radiation on human NSCLC cell lines correlated with the level of EGFR expression [56], and in a recent report of a phase II clinical trial, a significant survival improvement was observed for patients with EGFR-positive tumors that were treated with Nimotuzumab [33]. It remains to be shown whether the EGFR expression level is a predictive marker of Nimotuzumab's clinical efficacy, in contrast to high affinity antibodies like Cetuximab, for which it has been shown that the EGFR expression level is not a predictive marker of clinical benefit [58].

It might be possible that the therapeutic ratio of some of the existing high-affinity anti-EGFR antibodies could be improved by “optimizing” (in this case—lowering) the affinity, although other factors such as the location of the binding epitope on the EGFR might play an important role as well, as discussed below. Another issue to take into account is that intermediate affinity anti-EGFR antibodies might provide clinical benefit only for a subset of patients bearing EGFR-overexpressing tumors.

#### Inhibition of ligand-dependent receptor activation

The crystal structures of the Fab fragments of five different antibodies in complex with extracellular domains of ErbB receptors revealed that, although they show distinct modes of binding, they have one thing in common, all of them, directly or indirectly, inhibit the receptor dimerization event that triggers the proliferative signaling [59]. Cetuximab, for example, binds to domain III of the EGFR and inhibits both EGF binding and the active conformation of the receptor monomer [53]. Panitumumab also binds to domain III, to a site that overlaps with Cetuximab's epitope and with the EGF binding site on this domain. Thus, it is highly likely that Panitumumab's mechanism of EGFR inhibition is similar to that of Cetuximab [59].

Recent experimental and computer modeling data indicate that Nimotuzumab might have a different mechanism of action. The epitope recognized by this antibody on the EGFR strongly overlaps with that of Cetuximab, as demonstrated by site-directed mutagenesis, but is slightly displaced towards the C-terminus of EGFR domain III, according to a computer model [55]. In this position, Nimotuzumab sterically interferes with EGF binding while permitting the receptor to adopt its active conformation and, in consequence, form a homo—or heterodimer—with a second molecule of the EGFR family. This way, Nimotuzumab would allow a certain level of basal, ligand-independent level of EGFR activation, needed for the survival of normal epithelial cells. On the other hand, the antibody would prevent the ligand-induced shift of the equilibrium towards the active conformation, having the effect of “freezing” the tumor growth. This mechanism would explain why Nimotuzumab produces mostly a cytostatic effect [60] and induction of stable disease [32]. Interestingly, recent results show that Nimotuzumab at a high concentration is able to weaken the ligand-independent signaling in cells with a high level of EGFR expression (*manuscript in preparation*). Bivalent binding may account for this effect, since at high EGFR concentrations the antibody would cause a pairwise receptor cross-linking, thereby preventing the formation of active EGFR dimers, as has been shown by high resolution electron microscopy for the antibody Zalutumumab [61].

#### Targeting of CD133+ cancer stem cells

In a study by Díaz-Miqueli and coworkers [62] using U187 human glioma tumor xenografts in nude mice, both Nimotuzumab and Cetuximab, in spite of their differences in cytotoxicity, induced a reduction in radioresistant CD133+ tumor stem cells, impairing tumor growth progression without producing a tumor shrinkage. It has been suggested that brain tumor growth is critically dependent on the presence of an intact cancer stem cell vascular niche [63]. Therefore, the ability to target cancer stem cells may represent an important property for an anti-EGFR antibody.

#### Anti-angiogenic effects

The anti-tumor activity of Nimotuzumab has been associated with anti-proliferative and anti-angiogenic effects rather than direct induction of tumor cell death [60]. We demonstrated several years ago that the induction of *in vivo* resistance to Nimotuzumab by A431 human tumor cells was mostly due to constitutive VEGF gene overexpression [64]. In more recent experiments by Diaz-Miqueli *et al.* [62], Nimotuzumab combined with radiotherapy produced a reduction in the size of tumor blood vessels and the number of proliferating cells in subcutaneous tumors. The mechanism by which Nimotuzumab achieves this effect remains unknown.

### 3.3. Immunomodulatory Effects of EGFR Targeting

#### Induction of anti-tumor cellular immune responses

Analysis of the clinical data obtained in several randomized trials with Nimotuzumab evidences a delayed separation of the survival curves, indicating a non-proportional hazard ratio between treated and control patients along the follow-up time [33]. A possible explanation for these results would be a time-delayed induction of a protective immunity. Using a syngeneic mouse model, we have demonstrated that treatment with an anti-murine EGFR-antagonistic antibody, called 7A7, increases the number of various immune cells in metastatic sites, in particular T lymphocytes and dendritic cells, which might be implicated in the development of an anti-tumor specific immune response. Indeed, depletion of CD4+ and CD8+ cells *in vivo* totally abrogated the anti-metastatic effect produced by the 7A7 mAb [65]. Recent findings indicate that this antibody induces an immunogenic apoptotic cell death in D122-3LL murine tumor cells [66].

Active immunotherapy approaches aimed to stimulate both humoral and cellular immune responses are also being tested. One of these vaccines, consisting of a peptide enclosing the tumor-specific mutated segment of EGFRvIII, conjugated to KLH, has been shown to elicit antibody and cellular immune responses in mice and in patients [67], and is at present in phase II clinical trials for malignant glioma. We are currently developing a cancer vaccine based on the extracellular region of the EGFR, adjuvated with a proteoliposome from the outer membrane *of Neisseria meningitidis* bacteria [68]. Preclinical studies in animal models were recently completed, and last year the HER1 vaccine candidate entered a phase I clinical trial in Cuba, in patients with hormone-refractory prostate cancer.

It has been argued that the “vaccinal effect” of anti-tumor monoclonal antibodies may have an important weight in their clinical benefit [69], as demonstrated for the anti-CD20 antibody Rituximab, which has been shown to elicit an active T cell response specific for follicular lymphoma [70]. But for anti-EGFR mAbs, to our knowledge, there are no clinical data showing an enhancement of anti-tumor cellular immune responses as result of a passive immunotherapy. Our mechanistic studies with the 7A7 antibody prompt us to measure anti-tumor specific cytotoxic T cell (CTL) responses in patients treated with anti-EGFR antibodies.

#### Impairment of tumor-induced immunosuppression

Oncogene activation has been associated with a down-regulation of the antigen processing machinery, making tumor cells “less visible” to CTLs [71] More recently, oncogene activation has also been related to the induction of an immunosuppressive microenvironment by tumor cells, via up-regulation of pro-inflammatory cytokine secretion [31]. Transcription factors like Stat3, NF-Кβ and HIF-1α link oncogene activation signaling pathways with the molecular mechanisms governing the cross-talk between tumor cells and the immune system. Therefore, EGFR-targeting would have a double impact—arresting tumor cell proliferation and impairing tumor immune evasion. The experimental demonstration of this hypothesis is very appealing because of its translational impact. In particular, it becomes important to take into account the immunomodulatory effects of anti-EGFR antibodies when designing new combination therapies [72]. On the other hand, different EGFR antagonists may have different effects on cancer-related inflammation, which also needs to be translated into differentiated strategies.

## 4. Conclusions

### 4.1. EGFR-Targeted Treatment as Biological Therapy

Targeted therapies have not yet provided the expected clinical benefit in advanced cancer patients: therefore new clinical strategies are needed. We believe that targeted-therapies have the potential to transform advanced cancer into a long-term controlled chronic disease, and EGFR-targeting may represent a suitable scenario to show that this approach is feasible. Objective clinical response is a signature of cytotoxic drugs as a consequence of tumor shrinkage, but on the other hand, it has been extensively documented that in most cases there is no correlation between objective clinical response and survival time, due to tumor recurrence. Targeted-therapies may instead be aimed to stop disease progression based on biological regulatory mechanisms.

The inhibition of EGF-dependent receptor activation would stop tumor progression by at least four different mechanisms: (1) arrest of tumor cell growth; (2) homeostatic regulation of tumor population dynamics; (3) reduction of the number of cancer stem cells; and (4) enhancement of the anti-tumor cellular immune response (Box 1).

Box 1. **Surpassing the cytotoxic paradigm in anti-EGFR therapies**

**EGFR-immunotargeting as a biological therapy**

- Arrest of tumor cell growth
- Reduction of cancer stem cells
- Homeostatic regulation of tumor population dynamics
- Activation of the adaptive immune system

**A tailored clinical evaluation strategy**

- Efficacy assessment assuming a non-proportional hazard ratio
- Treatment beyond early progression
- Chronic use (long-term schedule)
- Combination therapies with metabolic inhibitors, anti-inflammatory drugs, immunomodulators

Because EGFR-overexpression is a hallmark of advanced epithelium-derived tumors, anti-EGFR antibodies bind preferentially to tumor cells. Nevertheless, binding to normal epithelial cells also occurs, causing toxic effects. An advantageous therapeutic ratio for a given anti-EGFR antagonistic antibody might be attained by adjusting the administration schedule based on pharmacodynamic studies. Such a treatment schedule could be used for long time periods without resulting in cumulative toxicity. Combination therapies and long-term treatment schedules, even after early disease progression, are currently being evaluated for Nimotuzumab.

## Figures and Tables

**Table 1.** Clinical benefit from FDA-approved anti-EGFR agents.

**Drug**	**Indication**	**Treatment**	**Clinical benefit**
Cetuximab	Locally or regionally advanced SCCHN	Radiation + Cetuximab *vs.* Radiation	Duration of loco-regional control: 24.4 *vs.* 14.9 months [11]
	Recurrent or metastatic SCCHN in progression after platinum based chemotherapy	Cetuximab monotherapy	RR: 13%. Duration of response: 5.8 months [12]
	Metastatic colorectal cancer refractory to Irinotecan and oxaliplatin based therapy	Cetuximab *vs.* BSC	MST: 6.14 *vs.* 4.57 months *1 [13]
	Metastatic colorectal cancer (Irinotecan refractory)	Cetuximab + Irinotecan *vs.* Cetuximab	RR: 23% *vs.* 11%. Duration of response: 5.7 *vs.* 4.1 months *,1 [14]
Panitumumab	Metastatic colorectal cancer with disease progression following fluoropyrimidine, oxaliplatin, and irinotecan regimens.	Panitumumab *vs.* BSC	PFS: 96 *vs.* 60 days *,1 [15]
Erlotinib	Locally advanced or metastatic NSCLC refractory to first or second line chemotherapy	Erlotinib *vs.* BSC	MST: 6.7 *vs.* 4.7 months [16]
	Locally advanced or metastatic NSCLC whose disease has not progressed after four cycles of platinum-based first-line chemotherapy	Erlotinib *vs.* Placebo	MST: 2.8 *vs.* 2.5 months [17]
	Locally advanced, unresectable or metastatic pancreatic cancer	Erlotinib + Gemcitabine *vs.* Gemcitabine	MST: 6.4 *vs.* 6.0 months [18]
Gefitinib	NSCLC refractory to first or second line chemotherapy	Gefitinib monotherapy	RR: 10.6% Duration of response: 7 months *,2 [19]
	NSCLC refractory to first line chemotherapy	Gefitinib *vs.* Docetaxel	MST: 7.6 *vs.* 8 months *,2 [20]
	Advaced NSCLC naïve for chemotherapy (Asian patients, never smoking, ADC and bronchoalveolar carcinoma)	Gefitinib *vs.* Carbo/Taxol	PFS: 5.7 *vs.* 5.8 months *,2 [21]

*Abbreviations:* BSC: best supportive care; MST: median survival time; NSCLC: non-small cell lung cancer; PSF: progression free survival; RR: response rate; SCCHN: squamous cell carcinoma of the head and neck;

*,1 The approval of cetuximab and panitumumab in colorectal cancer was later amended to include only patients with wild-type KRAS;

*,2 The approval of gefitinib in NSCLC was later amended to include only patients who, in the opinion of their treating physician, are currently benefiting, or have previously benefited, from gefitinib treatment.

**Table 2.** Clinical benefit from controlled clinical trials with Nimotuzumab.

**Indication**	**Trial Design**	**Clinical Benefit [References]**
Advanced SCCHN	Nimo + RTP 24 pts, 6 doses, 200 mg, weekly	MST: 45.2 months [43]
	Nimo + RTP/CTP *vs.* RTP/CTP 46 patients, 6 doses, 200 mg, weekly	RR (at 24 weeks): 100% *vs.* 70% (p = 0.02) SV rate (30 months): 69.6% *vs.* 21.7% (p = 0.0011) [44]
	Nimo + RTP *vs.* RTP 46 patients, 6 doses, 200 mg, weekly	RR (at 24 weeks): 76% *vs.* 40% (p = 0.023) RR(at 30 months): 39.1% *vs.* 21.7% (p = 0.2) [44]
	Nimo + RTP *vs.* placebo + RTP 106 patients, 6 doses, 200 mg, weekly	CRR: 59.5% *vs.* 34.2% (p = 0.038) [33]
Advanced nasopharyngeal cancer	Nimo + RTP *vs.* RTP 137 patients, 6 doses, 100 mg, weekly	CRR: 90.63 % *vs.* 51.52% (p < 0.05) [45]
Relapsed childhood glioma	Nimo monotherapy, 47 patients, 150 mg/m^2^, 6 doses weekly and maintenance bi-weekly until PD	DCR: 38.1% [46]
Childhood glioma	Relapsed glioma: Nimo monotherapy, 37 patients.Newly diagnosed: Nimo + RTP + vinorelbine, 10 patients 150 mg/m^2^, 6 doses weekly and maintenance bi-weekly until PD	MST: 11 months PFS 6 months: 90% [38]
High grade glioma (adults)	Nimo + RTP, 29 patients 6 doses, 200 mg, weekly	MST (GBM): 17.47 months [40]
Advanced or recurrent gastric cancer	Nimo + irinotecan *vs.* irinotecan 82 patients, 6 doses,400 mg, weekly	MST: 293 *vs.* 227 days, HR 0.717, (p = 0.42) EGFR 1+/2+/3+ OS HR 0.584 (p = 0.242) EGFR 2+/3+ OS HR 0.295 (p = 0.077) [47]
NSCLC (Unfit for radical therapy)	Nimo + palliative RTP, 17 patients 6 doses, 100–400 mg, weekly and maintenance bi-weekly until PD	DCR: 94% [48]
	Nimo + palliative RTP, 15 pts, 6 doses, 100–400 mg, weekly and maintenance bi-weekly until PD	DCR: 100% [49]

*Abbreviations:* MST: median survival time; RR: response rate (complete and partial response); SV rate: survival rate; CRR: complete response rate; DCR: disease control rate (complete and partial response plus stable disease); PFS: progression free survival; OS: overall survival; HR: hazard ratio; PD: progressive disease; RTP: radiotherapy; CTP: chemotherapy.
